# The Role of Mast Cell Specific Chymases and Tryptases in Tumor Angiogenesis

**DOI:** 10.1155/2015/142359

**Published:** 2015-06-04

**Authors:** Devandir Antonio de Souza Junior, Ana Carolina Santana, Elaine Zayas Marcelino da Silva, Constance Oliver, Maria Celia Jamur

**Affiliations:** Department of Cell and Molecular Biology and Pathogenic Bioagents, Ribeirão Preto Medical School, University of São Paulo, 14049-900 Ribeirão Preto, SP, Brazil

## Abstract

An association between mast cells and tumor angiogenesis is known to exist, but the exact role that mast cells play in this process is still unclear. It is thought that the mediators released by mast cells are important in neovascularization. However, it is not known how individual mediators are involved in this process. The major constituents of mast cell secretory granules are the mast cell specific proteases chymase, tryptase, and carboxypeptidase A3. Several previous studies aimed to understand the way in which specific mast cell granule constituents act to induce tumor angiogenesis. A body of evidence indicates that mast cell proteases are the pivotal players in inducing tumor angiogenesis. In this review, the likely mechanisms by which tryptase and chymase can act directly or indirectly to induce tumor angiogenesis are discussed. Finally, information presented here in this review indicates that mast cell proteases significantly influence angiogenesis thus affecting tumor growth and progression. This also suggests that these proteases could serve as novel therapeutic targets for the treatment of various types of cancer.

## 1. Introduction

Angiogenesis is a dynamic process mediated by endothelial cells whereby new blood vessels are formed from existing ones [1, 2]. Angiogenesis is crucial during physiological processes such as embryonic development and corpus luteum formation, and it is also involved in the development of pathological conditions such as tumorigenesis and chronic inflammation [3, 4]. This process is highly regulated by the balance between proangiogenic and antiangiogenic factors within the vascular microenvironment and involves the participation of extracellular matrix (ECM) proteins, adhesion molecules, and proteolytic enzymes [5, 6]. The main proangiogenic factors include vascular endothelial growth factor (VEGF), fibroblast growth factor (FGF), transforming growth factor-beta (TGF-*β*) platelet-derived growth factor (PDGF), interleukin-8 (IL-8), and angiopoietin-1 [7, 8]. A change in the balance between proangiogenic and antiangiogenic factors towards the activation of usually quiescent endothelial cells initiates a series of sequential events that characterize the angiogenic process, that is, ECM degradation, migration and proliferation of endothelial cells, formation of tube-like structures on 2D matrices and sprouting in 3D matrices, spatial distribution of newly formed vessels, deposition of new ECM, and mobilization of pericytes for vessel stabilization, and finally a shift in the balance towards inhibition of angiogenesis returns the endothelial cells to their quiescent state [9–12].

The importance of angiogenesis for tumor development has long been investigated. In the beginning of the 20th century, Goldmann [13] had already envisioned the importance of the vascular system for tumor growth. However, it was not until 1971 that Folkman [14] showed that tumor growth was dependent on angiogenesis and proposed the use of antiangiogenic therapy against cancer. Today, tumor angiogenesis is considered crucial for cancer development through induction of tumor growth, invasion, and metastasis [15, 16]. Moreover, the use of antiangiogenic agents as adjuvants for cancer treatment has become a reality [17].

Tumor angiogenesis relies on the abnormal switch in the balance between positive and negative regulators, usually triggered by the release of angiostimulatory mediators from neoplastic and inflammatory cells. The binding of these mediators to their receptors on endothelial cells activates the angiogenesis cascade [18–20]. The pro- and antitumorigenic signals provided by tumor cells and resident and recruited immune cells are ultimately responsible for the outcome of tumor progression. These signals act in processes such as proliferation and survival of tumor cells, angiogenesis, immune system modulation, tissue remodeling, and metastasis [21]. Therefore, the interaction between tumor, stromal, and immune cells collaborates to establish a “proangiogenic/tumorigenic” microenvironment favoring the survival, development, and spreading of tumor cells [22].

The view that immune cells are critical in providing the angiogenic stimulus required for tumor growth and progression is supported by a number of studies assigning proangiogenic roles to various innate immune cell types, in particular macrophages and mast cells [23]. In this review article, the importance of mast cells in tumor angiogenesis with special reference on the role of mast cell proteases in this process will be discussed.

## 2. Mast Cell Biology

Mast cells are bone-marrow-derived multifunctional immune cells implicated in both physiological and pathological settings (reviewed in [24]). Mast cell progenitors leave the blood circulation to mature and reside in the connective tissue, at the interface between the host and the outside environment, predominantly in subepithelial regions and in the connective tissue surrounding blood vessels, nerves, lymphatic vessels, and mucus glands [25, 26]. Mast cell distribution relies on mechanisms of constitutive homing, enhanced recruitment, survival, and local maturation of mast cell progenitors. In physiological conditions, their numbers remain relatively constant; however, proliferation and the enhancement of the mechanisms described above lead to mast cell hyperplasia during hypersensitivity reactions and infections and in response to various disease processes [27–29].

Mast cells' functions are a direct consequence of the diversity of biologically active compounds that they are able to produce and secrete. Upon activation, mast cells can release a wide range of mediators belonging to three distinct classes: preformed mediators, which are stored in mast cell cytoplasmic granules; neoformed or lipid mediators, which are products of phospholipase A2 activation; and neosynthesized mediators, which are produced and released upon transcriptional activation [25].

Mast cells may be activated by distinct stimuli acting on the various surface receptors expressed by these cells. Moreover, the variety of mast cell responses to different stimuli, that is, the nature of the mediators released, is influenced by mast cell heterogeneity. This heterogeneity is ultimately dictated by the intrinsic and microenvironmental factors that these cells encounter in normal and pathological conditions [25, 30].

A distinct feature of mature mast cells is the high content of metachromatic, electron dense secretory granules present in their cytoplasm. These secretory granules store an extensive variety of preformed bioactive mediators, which are all stored in the active form and are promptly released by degranulation. Mast cell degranulation can be triggered by various mechanisms such as the crosslinking of Fc*ε*RI-bound IgE by specific antigens, crosslinking of Fc*γ*RIII by IgG immune complexes, complement receptor engagement by complement components such as C3a and C5a, c-kit receptor binding by stem cell factor (SCF), TLR2 (toll-like receptor 2) stimulation, or by binding of neuropeptides, and other peptides [31–37]. The preformed mediators include, but are not restricted to, biogenic amines, lysosomal enzymes, proteases, proteoglycans, cytokines, chemokines, growth factors, and peptides [31, 33, 36–38].

The major constituents of mast cell secretory granules are the mast cell specific proteases chymase, tryptase, and carboxypeptidase A3 (CPA3) (Table 1) [39]. These proteases are exclusively expressed by mast cells and they represent approximately 25% of the cell protein content. They are stored in their active form within the secretory granules [40–42]. The tryptases and chymases are serine proteases while CPA3 is a zinc-dependent metalloprotease [39, 43].

Like other positively charged preformed mediators, mast cell proteases are efficiently packed into mast cell secretory granules owing to their interaction with negatively charged serglycin proteoglycans [43, 44]. However, not all subtypes of mast cell proteases are dependent on serglycin for storage. A study using knockout mice for serglycin core protein showed that although the protease mRNA levels were similar between knockout and wild type mice the interaction of mast cell proteases with serglycin was necessary to regulate the storage of mouse mast cell protease- (mMCP-) 4, mMCP-5, mMCP-6, and CPA in the granules [45]. On the other hand, Braga et al. [46] showed that the storage of mMCP-7 and mMCP-1 was independent of serglycin. Moreover, Melo et al. [44] have recently shown that serglycin dependent storage of mast cell proteases is critical in the induction of apoptosis induced by permeabilization of the granule membrane.

The mast cell protease content varies according to the tissue distribution of mast cells as well as from species to species, and these differences are used to phenotypically classify mast cells (Table 1) [47]. Human mast cells are divided into MC_T_ that express tryptases *α* and *β* and MC_TC_ that express chymases, tryptases, and CPA3. A third phenotype expressing tryptases and CPA3 was recently described in airway epithelium and esophageal samples of patients with asthma and eosinophilic esophagitis [48, 49]. In rodents, mast cells are classified according to their distribution as connective tissue mast cells (CTMCs), which express chymases (*α* and *β*), tryptases, and CPA, and mucosal mast cells (MMCs), which express the *β* chymases, mMCP-1 and mMCP-2. It is noteworthy to mention that these mouse mast cell phenotypes can vary significantly according to mast cell location, animal strain, and whether or not the tissue is inflamed [50–53]. Mast cell proteases have been implicated in a number of pathological states including arthritis, allergic airway inflammation, and tumor angiogenesis [54–59].

## 3. Mast Cells, Tumors, and Angiogenesis

The association between mast cells, inflammation, and cancer is conflicting and involves both promotion of and protection against tumor progression. The first association of mast cells with tumors dates back from the initial description of mast cells by Ehrlich in 1878, when he reported that mast cells were numerous in some tumors [60]. Since then, interest in the contribution of mast cells to tumor development has increased progressively. Mast cells have been shown to accumulate around several types of tumors and are generally the first inflammatory cells to infiltrate developing tumors such as malignant melanoma and breast and colorectal tumors [61–64]. This accumulation typically occurs around blood vessels within the tumor environment and correlates with both good and poor prognosis in different cancers demonstrating the paradoxical involvement of mast cells in tumor progression [65–67]. Mast cells are recruited by several tumor-derived factors including the angiogenic factors VEGF, PDGF, and FGF-2 [68]. Notably, Huang et al. [69], using an hepatocarcinoma model, showed that mast cells were unable to migrate in SCF-knockout tumors or in the presence of anti-c-kit antibodies, ultimately resulting in decreased tumor growth. In addition, SCF stimulation leads to the release of matrix metalloproteinase-9 (MMP-9), IL-6, TNF-*α*, VEGF, Cox2, i-NOS, and chemokine (C-C motif) ligand 2 (CCL2). These results suggest that mast cell recruitment and activation, with the consequent release of inflammatory mediators that participate in tissue remodeling and immune suppression, are mainly mediated by SCF and its receptor c-kit expressed on the mast cell surface. The contribution of mast cells to the promotion of cancer includes their action on immunosuppression, the release of proangiogenic and mitogenic factors, and degradation of the ECM [61].

Mast cells have been shown to be associated with angiogenesis and tumor progression in numerous types of cancer [65, 70–80]. Stimulation of angiogenesis is undoubtedly the most significant role of mast cells in the promotion of tumor growth and development [81]. In fact, mast cell deficient mice showed a reduced angiogenesis and metastatic capacity during tumor induction [82]. The involvement of mast cells in these processes is most certainly related to the release of a plethora of potent proangiogenic mediators which include histamine, angiopoietin-1, VEGF, FGF-2, TGF-*β*, tumor necrosis factor-alpha (TNF-*α*), and IL-18 [81, 83, 84]. Furthermore, a role for mast cell specific proteases has gained increasing support [74, 85–89]. For instance, mast cell specific proteases are involved in the initial phases of tumor growth and also modulate vascular growth in the later stages of tumor progression [59]. Mast cell mediators can induce angiogenesis through their action at different stages of angiogenesis, that is, degradation of the ECM, migration and proliferation of endothelial cells, formation and distribution of new vessels, synthesis of ECM, and pericyte mobilization [10, 90].

Most angiogenic mediators released by mast cells are not exclusive to mast cells, so it is difficult to attribute their biological actions to mast cells alone. Therefore, the majority of recent investigations on the role of mast cells in tumor angiogenesis have focused on the ability of mast cells to synthesize, store, and release mast cell specific chymases and tryptases. The role of these proteases in tumor angiogenesis is the subject of this review.

## 4. Tryptases in Tumor Angiogenesis

Mast cell tryptase is a tetrameric neutral serine protease with a molecular weight of 134 kDa [54, 91]. Mast cells contain 10–35 pg tryptases per cell [92]. The enzyme consists of four noncovalently bound subunits. Each subunit has one active catalytic site and disruption of the tetramer into monomers causes inactivation of the tryptase [40, 93]. Tryptases were named because of their trypsin-like substrate specificity (Table 1). They cleave preferentially after Lys/Arg residues [39, 94]. Humans express two classes of tryptase (alpha and beta). Alpha-tryptase is the major circulating isoform and *β*-tryptase appears to be the major type stored in secretory granules [54, 94–96]. Three similar *β*-tryptases (*β*I–III) and one *α*-tryptase have been identified. The *β*-tryptases are 98-99% identical in amino acid sequence, while the *α*-tryptase is less closely related. The mast cell tryptases (MCP-6 and MCP-7) expressed in rodents most likely represent the counterparts to human *α*-tryptase and *β*-tryptases, respectively. Additionally, both subtypes are initially expressed as monomers in humans and rodents [95, 96] and then combine to form tetramers.

The first reports on the association between tryptase and tumor angiogenesis focused on the use of tryptase as a specific marker for mast cells. Kankkunen and colleagues [97] were among the first to study this relationship. They analyzed the number and distribution of tryptase- and chymase-positive mast cells in benign and malignant breast lesions [97]. Their results show that the number of tryptase-positive mast cells in malignant tumors was increased four times in relation to the benign lesions. Moreover, in malignant lesions, tryptase-positive mast cells were concentrated at the tumor edge within the “invasion zone.” Since then several other studies have corroborated this correlation between tryptase and malignant tumors in various types of tumor, such as lung adenocarcinoma [98]; B-cell non-Hodgkin's lymphomas [80], brain [99] and cervical cancer [70], myelodysplastic syndrome [4], B-cell chronic lymphocytic leukemia [100], melanoma [101], hepatocellular carcinoma [102], gastrointestinal cancer [103], early breast cancer [104], and colorectal cancer [105]. During the last two decades, several studies, using tryptase as a mast cell marker, have shown that mast cells are strategically located in proximity to blood vessels where they have been associated with tumor angiogenesis. These studies on the correlation between tryptase-positive mast cells and angiogenesis also showed a positive association between tumor aggressiveness and poor prognosis [106–108].

Tryptases act on a variety of substrates [39, 96]. Processing of several of these substrates are known to be key factors in angiogenesis, such as degradation of extracellular matrix [109, 110], plasminogen activation [111], fibrinogen degradation [112, 113], activation of latent collagenase and matrix metalloproteinases [114, 115], and tryptase-induced endothelial barrier dysfunction [116, 117]. The correlation between the presence of tryptase-positive mast cells and angiogenesis as well as the action of tryptase on angiogenesis related substrates supports the hypothesis that the presence of tryptase is critical for the initiation of angiogenesis. In spite of the fact that these studies only showed the presence of mast cells at angiogenic sites, they provided a basis for further investigation on the direct relationship between tryptase and angiogenesis.

Blair et al. [118] were the first to investigate the potential of tryptase to induce* in vitro* angiogenesis using a tube formation angiogenesis assay. This assay involves plating endothelial cells on a gel matrix and assaying adhesion, migration, and formation of capillary or tube-like structures. These capillary-like structures consist of tubes and loops. Tubes are formed by endothelial cell sheets and go from one branch point to another branch point or to a loose end. Loops are the enclosed (or almost enclosed) spaces inside the tubes. In these experiments, they incubated human dermal microvascular endothelial cells (HDMECs), plated in matrigel-coated wells, in the presence of purified human lung tryptase for 16 hours. The tryptase induced an increase in capillary-like structures and proliferation of HDMECs. This effect was suppressed by the use of protease inhibitors. These results lead to the conclusion that tryptase interacts directly with endothelial cells via an unidentified mechanism to induce angiogenesis.

Compton et al. [119] also investigated the ability of tryptase to induce* in vitro* angiogenesis. However, in this study tryptase did not stimulate growth of human umbilical vein endothelial cells (HUVEC). They observed that tryptase induced the release of IL-8 in a dose-dependent manner. They also confirmed that this effect on HUVEC required enzymatically active tryptase. Release of IL-8 did not occur in the presence of heat inactivated enzyme or when the tryptase was preincubated with the protease inhibitors.

Using a mouse model of epithelial carcinogenesis, Coussens et al. [120] observed that, during the dysplasia stage, vascular changes indicative of an angiogenic switch from vascular quiescence to modest neovascularization occurred in early modest to low-grade lesions [120]. At this stage, the researchers observed a significant increase in tryptase activity and mMCP-6 mRNA expression. In this same study, mMCP-6 did not directly affect the extracellular matrix, but mMCP-6 acted indirectly through the stimulation of procollagen synthesis by fibroblasts. The results of this elegant study indicate that tryptase can affect the extracellular matrix by stimulating the cellular components, thus altering its composition that then leads to neovascularization. Additionally, other studies have demonstrated the ability of tryptase to digest isolated extracellular matrix [109, 121, 122]. Further studies have shown that tryptase can also activate metalloproteinases thus inducing degradation of the extracellular matrix [114, 123]. Taken together, these results suggest that tryptase may have both a direct and indirect action in the remodeling and degradation of the extracellular matrix during angiogenesis. The role of tryptase in degradation/remodeling of the extracellular matrix may vary depending on the type of tumor and phase of tumor progression. This process of degradation/remodeling could increase the space available for neovascularization and contribute to the release of angiogenic factors present in the matrix such as VEGF and FGF-2 [113, 124].

Another direct effect of tryptase in various cell types, including endothelial cells, is stimulation of protease activated receptor-2 (PAR-2) [125, 126]. Yoshii and coworkers [74] studying the direct effects of tryptase on the proliferation and intracellular signaling in DLD-1 colorectal adenocarcinoma cells* in vitro* demonstrated that tryptase induces proliferation in DLD-1 cells through its proteolytic activity on PAR-2. The increase in proliferation was mediated in a cyclooxygenase- and MAP kinase-dependent manner. Recently, Zhi and colleagues [127] demonstrated* in vitro* that tryptase increased bEnd.3 mouse brain endothelial cell proliferation, migration, and tube formation, suggesting a role for tryptase in microvessel formation. They also observed that tryptase increased tissue plasminogen activator (tPA) and inhibited plasminogen activator inhibitor-1 (PAI-1) expression at both mRNA and protein levels in bEnd.3 cells.

Therefore, tryptase, through the activation of PAR-2, may have a role in modulating the balance between expression of urokinase plasminogen activator (uPA) and PAI-1. Expression of uPA and PAI-1 is PAR-dependent [128, 129]. Additionally, PAR-2 activation in vascular cells promotes angiogenesis during tissue repair as well as during neovascularization of the retina [130, 131]. PAR-2 also induces endothelial cell proliferation [132, 133] and cytokine expression in endothelial cells and other cell types [134–136]. Together these studies indicate that the activation of PAR-2 by tryptase is directly related to the role of mast cells in angiogenesis. The exact mechanism by which PAR-2 contributes to angiogenesis remains to be elucidated. Nevertheless, the involvement of tryptase in PAR-2 activation and angiogenesis is clear.

**Figure 1:**
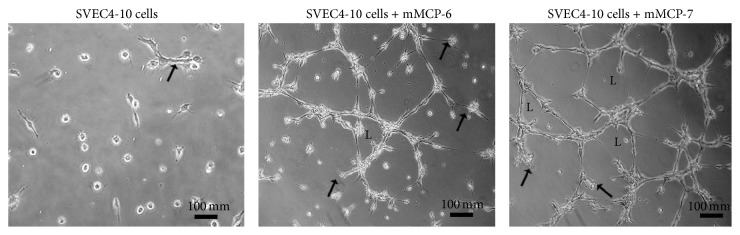
rmMCP-6 and rmMCP-7 induce tube formation by mouse endothelial cells.

Tryptase (mMCP-6 and mMCP-7) expression and activity were increased during progression of skin tumors in mice [59]. Furthermore, the increase in tryptase activity was correlated with the tumor angiogenesis. mMCP-7 was not detectable in control animals but became measurable concomitantly with the initiation of angiogenesis during tumor progression. The ability of these two subtypes of tryptase to induce angiogenesis* in vitro* was compared. mMCP-6 and mMCP-7 were both able to induce tube formation in SVEC4-10 endothelial cells. However, mMCP-7 was more efficient in inducing cell spreading and tube formation than mMCP-6 (Figure 1). These data show that both subtypes of mast cell specific tryptases may interact with endothelial cells to promote neovascularization of tumors.

When endothelial cells were incubated in absence of tryptases a few SVEC4-10 cells are spread and an occasional tube is seen (arrow). When the SVEC4-10 cells were incubated with rmMCP-6, cells are spread, and tubes (arrows) and loops (L) are present. When the SVEC4-10 cells were incubated with rmMCP-7 tubes (arrows) and loops (L) are more prevalent. Endothelial cells (SVEC4-10) were cultivated in wells of *μ*-slides angiogenesis (ibidi GmbH, Martinsried, Germany) with Geltrex (Thermo Fisher Scientific Inc. Waltham, MA) for 5 hours at 37°C in the presence or absence of the subtypes of tryptase. Following incubation, the samples were fixed, viewed, and photographed using a Nikon Eclipse TE2000-U (Nikon Instruments Inc., Melville, NY) phase contrast inverted microscope equipped with a Nikon DXM-1200 digital camera.

Recently, Wang et al. [137] examined the involvement of mast cells in angiogenesis* in vitro* by coculturing myocardial microvascular endothelial cells (MMVEC) with rat peritoneal mast cells (activated or nonactivated) or with only the granules from rat peritoneal mast cells. Mast cells activated by 48/80 induced* in vitro* angiogenesis, but this was not detected with nonactivated mast cells. To confirm if the mast cell granules (MCG) alone can induce* in vitro* angiogenesis, MMVEC were cultivated with MCG. There was significantly higher migration, proliferation, and capillary tube-like formation in the presence of MCG than without MCG. In addition, in the presence of MCG a decrease in Angiopoietin-2 (Ang-2) protein expression and mRNA by endothelial cells was seen, indicating that the MCG had a suppressive effect on Ang-2 expression. In order to determine if tryptase or chymase was responsible for the induction of angiogenesis via Ang/Tie-2, myocardial microvascular endothelial cells were cultivated with MCG in the presence of tryptase or chymase inhibitors. The ability of MCG to induce angiogenesis could be reversed by either tryptase or chymase inhibitors, although the tryptase inhibitor was more effective. Therefore, tryptase may be more important than chymase in inducing angiogenesis. Also tryptase may be the major factor released by mast cells that promotes neovessel formation. Taken together the results indicate that tryptase affects angiogenesis in MMVEC via Ang/Tie-2.

**Figure 2:**
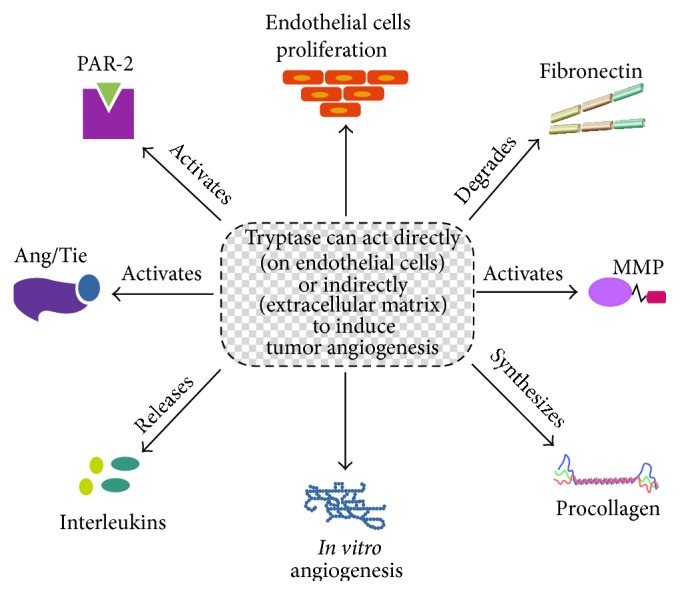
Tryptase can act directly or indirectly to induce tumor angiogenesis.

Since tryptase is known to be involved in tumor angiogenesis (Figure 2), recently it has been suggested that tryptase may serve not only as a biomarker in different pathologies but also as a promising therapeutic target. Ammendola et al. [103] review the possible molecular mechanisms of three drugs targeting tryptase and discuss their possible role in cancer therapy. Furthermore, the authors suggest mast cell tryptase as potential antiangiogenic strategy.

## 5. Chymases and Tumor Angiogenesis

Chymases are mast cell specific proteases that belong to the family of serine proteases. Mast cell chymases are monomeric endopeptidases [138] that have chymotrypsin-like specificities (Table 1). They preferentially cleave proteins after aromatic amino acid residues, except rat and mouse chymase 5 (rMCP-5 and mMCP-5), which have elastase-like rather than chymotrypsin-like substrate specificity [139]. Different chymases have been identified in various species and have been divided into two groups (*α*- and *β*-chymases) according to their structure and substrate specificity [140, 141]. Both subgroups of chymases convert Angiotensin I to Angiotensin II, but only *β*-chymases degrade Angiotensin II. The *α*-chymases cleave the Phe^8^-His^9^ bond of Angiotensin I [141] and are widely expressed in mammalian mast cells. The *β*-chymases can convert Angiotensin I to Angiotensin II but preferentially hydrolyze the Tyr^4^-Ile^5^ bond of Ang I and Ang II, yielding inactive peptide fragments [142].

Many other chymase substrates have been reported such as procollagen [143], pro-matrix metalloproteinase-9 (pro-MMP-9) [144, 145], tissue inhibitor of metalloproteinase-1 (TIMP-1) [146], big-endothelin-1 and big-endothelin-2 [147, 148], TGF-*β*1 [149], and thrombin [150]. However, chymases are relatively promiscuous in terms of cleavage specificity, and they have the capacity to cleave a multitude of substrates and can even cleave the same substrate at multiple positions.

In mast cells, chymases are stored as active enzymes in a macromolecular complex together with heparin in secretory granules. Prior to secretion, mast cell chymases are activated by dipeptidyl peptidase I [151]. Once secreted into the extracellular fluid, chymases remain in a complex with heparin proteoglycan, which protects the chymases from inhibition by endogenous inhibitors of chymase present in the extracellular fluid [152].

In man chymases are found in mast cells around blood vessels, in the heart, and many other tissues [153], but not in plasma [138]. The fact that chymases are present in different tissues and that they cleave numerous substrates indicates that chymases can play many different roles in physiological and pathological conditions, including tumor angiogenesis, but these roles are not well characterized. Additionally, the distribution of mast cells and mast cell subtypes is variable depending on the type of tumor. In colorectal cancer, hepatocellular carcinoma, and intrahepatic cholangiocarcinoma, both MC_TC_ and MC_T_ proliferate or infiltrate in the tumor [154, 155]. In contrast, in some malignant tumors, a significant increase in mast cell number was found. This observation was due to an increase in MC_T_ at the invasion zone, whereas the number of MC_TC_ remained constant in this area [70, 156].

**Figure 3:**
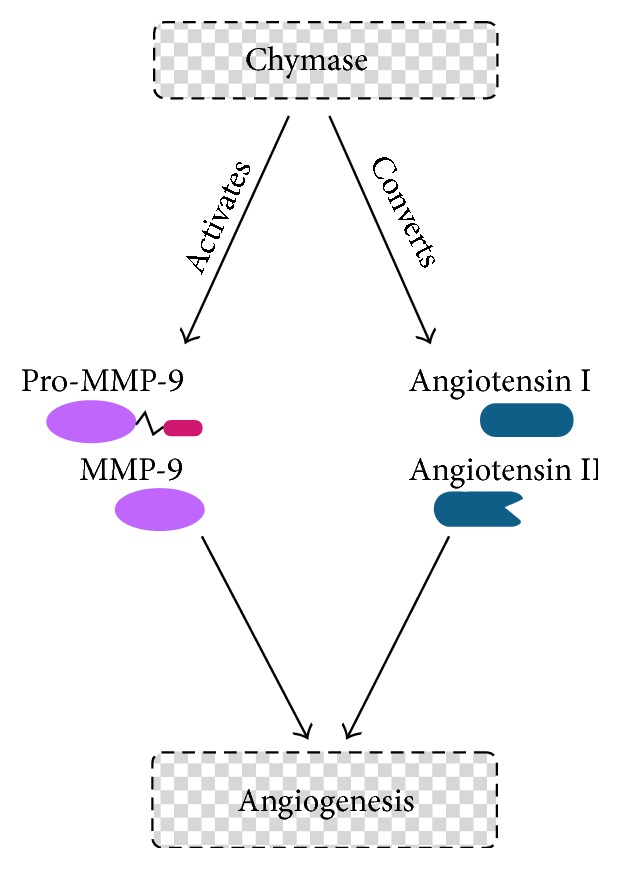
Chymase acts indirectly to induce angiogenesis. Chymase can activate MMP-9 or convert Angiotensin I to Angiotensin II.

Muramatsu et al. [157], using a hamster sponge implant model of angiogenesis, suggested a possible involvement of chymase in angiogenesis. Both transfection of human prochymase cDNA and injections of purified chymase into implanted sponges demonstrated that chymase was a powerful angiogenic factor. The authors then went on to investigate chymase as an alternative Angiotensin II-generating enzyme in angiogenesis. Chymase inhibitors reduced mast cell mediated angiogenesis in hamster sponge implant model [86, 158]. Furthermore, chymostatin (a chymase inhibitor) and TCV-116 (an Angiotensin II (Ang II) type 1 receptor agonist) completely inhibited bFGF-induced angiogenesis [85]. These results suggest that hamster chymase generates Angiotensin II from Angiotensin I, triggering bFGF-induced angiogenesis (Figure 3). Additionally, chymase mRNA expression was detected in bFGF-induced angiogenesis, but not in the control hamsters treated with saline. The increased angiogenesis was accompanied by a considerable increase in chymase activity in the granuloma tissues [86].

Local injection of compound 48/80, a mast cell activator, also promoted angiogenesis in hamster sponge granulomas through a chymase-dependent mechanism [85] and enhanced VEGF mRNA expression [159]. Treatment with neutralizing antibody against VEGF or an antisense oligodeoxynucleotide against VEGF supressed Angiotensin II-induced angiogenesis, showing that VEGF is a downstream factor in Angiotensin II-induced angiogenesis [159]. Therefore, the chymase-Ang II-VEGF pathway in granulation tissue may be the primary functional mediator of angiogenesis.

Arbeit et al. [160] developed a mouse model of epithelial carcinogenesis, by targeting the early region of human papillomavirus type 16 (HPV16) to the basal cells of the squamous epithelium using a human keratin 14 (K14) enhancer/promoter. With time after birth, these mice develop epidermal hyperplasia, which advances to angiogenic dysplasias. After one year, these dysplasias can progress to invasive squamous cell carcinomas [120]. In this transgenic mouse model an increase in mast cell number was observed during progression of the disease, especially in dysplasia [120], which is characterized by increased angiogenesis. At the same time, there was a significant increase in chymase activity and mMCP-4 mRNA was detected in dysplastic skin. Finally, hyperplastic ear skin from the transgenic mice was incubated in medium containing mMCP-4 and subsequently embedded in a collagen gel containing randomly dispersed bovine capillary endothelial cells (BCEs). The BCEs showed a dramatic response to the presence of skin that had been preincubated with mMCP-4. The endothelial cells became radially aligned, had increased rates of proliferation and migration, and exhibited tube formation toward the dermal surface, but not the epidermal surface of the skin pieces. However, the addition of mMCP-4 directly to BCEs did not induce a response, indicating that the angiogenic activity came from sequestered factors in the skin pieces used in this assay. Furthermore, canine and human chymases can cleave pro-MMP-9 producing active MMP-9. This matrix metalloproteinase participates in extracellular matrix remodeling [161] and regulation of angiogenesis [162]. In order to verify that mMCP-4 contributes to angiogenesis by activating MMP-9, hyperplastic skin lysates (containing predominantly pro-MMP-9) were incubated with mMCP-4 and then subjected to gelatin-substrate zymography. A substantially increased amount of active MMP-9 was detected, confirming that mMCP-4 plays a role in angiogenesis by activating MMP-9 thus inducing extracellular matrix remodeling [120]. These results, taken together, suggest that mMCP-4 acts indirectly to induce angiogenesis by activating pro-MMP-9, thus releasing sequestered angiogenic factors (Figure 3).

MCP-5 is emerging as a key player in chymase induced angiogenesis. The level of rMCP-5 mRNA in the granulomatous tissue of *λ*-carrageenin-soaked sponge implants was significantly increased in comparison to control granulomas [163]. Furthermore, angiogenesis during granuloma formation could be blocked by chymostatin and by blocking rMCP-5 with a specific antisense oligonucleotide. Recently, de Souza Jr. et al. [59] reported increased mast cell and blood vessel numbers as well as blood vessel diameter during mouse skin tumor progression. mMCP-5 expression was not detected in the control animals but was present in all stages of tumor progression. Total chymase activity and mMCP-5 expression increased during tumor progression and were correlated with tumor angiogenesis. In contrast, although mMCP-4 expression could be detected in control animals and in all phases of tumor progression, there was no difference in expression at the various phases.

The majority of the studies attempting to define the role of specific mast cell proteases, such as chymase in tumor angiogenesis in humans, have been focused on a pathological examination of tumor biopsies. The stage of the tumor, mast cell numbers, and mast cell subtype, that is, MC_TC_ and MC_T_, were all analyzed [78, 80, 156, 164–167]. These investigations have suggested a relation between chymase and angiogenesis in tumors. However, research into the chymase-containing mast cell subpopulations has been hampered by lack of suitable reagents.

In small sized adenocarcinomas of the lung, the mast cell number is increased in comparison with normal lung tissue and the ratio of MC_TC_ to total mast cells in the adenocarcinomas is significantly higher than that in normal lung. Furthermore, the accumulation of MC_TC_, but not MC_T_, correlates with a poor prognosis for patients with small sized lung adenocarcinoma. However, the numbers of both mast cell types correlated significantly with microvessel counts in small sized lung adenocarcinomas [168].

Mast cells are present in all three developmental phases of hemangiomas, which are benign, usually self-involuting tumors of the microvasculature [169]. In the initial proliferative phase, angiogenesis in hemangiomas is excessive but is followed by spontaneous regression, and the cellular parenchyma is gradually replaced with fibrofatty tissue. The mast cell number is the highest in the involuting phase, reduced in the involuted phase, and the least in the proliferative phase. However, the proportion of MC_TC_ decreases from the proliferative phase through the involuting phase to the involuted phase, indicating a proangiogenic role for chymase in this tumor. At the later stages, when the proportion of MC_T_ is increasing, these mast cells may be releasing tumor inhibitors.

In conclusion, the studies presented here support a relationship between mast cell chymases and tumor angiogenesis (Figure 3). However, additional studies are needed in order to elucidate the precise role of mast cell chymases in angiogenesis.

## 6. Conclusion

This review has presented evidence that tryptase and chymase act directly (on endothelial cells) or indirectly (on the extracellular matrix) to induce tumor angiogenesis. Most importantly, tryptase appears to play a more significant role in tumor progression than chymase. These findings support the use of mast cell proteases as novel tumor biomarkers as well as targets for antiangiogenic therapy. Furthermore, studies using chymase or tryptase knockout animals are necessary to demonstrate the functional impact of either chymase or tryptase on tumor angiogenesis.

## Figures and Tables

**Table 1:** Characteristics of major human and murine mast cell proteases.

Protein designation	Mast cell subtype	Chromosomal location	Gene name	Primary substrate specificity	Subunit structure
Chymase					

Human					
Chymase (CMA1)	MC_TC_	14q11.2	*CMA1 *	Chymotrypsin-like (aromatic amino acid)	Monomer

Mouse					
mMCP-1	MMC	14C1/2	*Mcpt1 *	Chymotrypsin-like (aromatic amino acid)	Monomer
mMCP-2	MMC	14C1/2	*Mcpt2 *	Unknown (low enzymatic activity)	Monomer
mMCP-4	CTMC	14C1/2	*Mcpt4 *	Chymotrypsin-like (aromatic amino acid)	Monomer
mMCP-5	CTMC	14C1/2	*Cma1/Mcpt5 *	Elastase-like (Val/Ala/Ile)	Monomer

Tryptase					

Human					
Tryptase *β*II/*β*III (human)	MC_TC_/MC_T_	16p13.3	*TPSB2 *	Trypsin-like (Arg/Lys)	Tetramer
Tryptase *α*I/*β*I (human)	MC_TC_/MC_T_	16p13.3	*TPSAB1 *	Trypsin-like (Arg/Lys)	Tetramer

Mouse					
mMCP-6	CTMC	17A3.3	*Mcpt6 *	Trypsin-like (Arg/Lys)	Tetramer
mMCP-7	CTMC	17A3.3	*Tpsab1 *	Trypsin-like (Arg/Lys)	Tetramer

Carboxypeptidase A3					

Human					
MC-CPA	MC_TC_	3q24	*CPA3 *	CPA-like (C-terminal aromatic/aliphatic amino acids)	Monomer

Mouse					
MC-CPA	CTMC	3A3	*Cpa3 *	CPA-CPA-like (C-terminal aromatic/aliphatic amino acids)	Monomer
